# The PAX8 cistrome in epithelial ovarian cancer

**DOI:** 10.18632/oncotarget.22718

**Published:** 2017-11-27

**Authors:** Emily K. Adler, Rosario I. Corona, Janet M. Lee, Norma Rodriguez-Malave, Paulette Mhawech-Fauceglia, Heidi Sowter, Dennis J. Hazelett, Kate Lawrenson, Simon A. Gayther

**Affiliations:** ^1^ Department of Preventive Medicine, University of Southern California/Keck School of Medicine, Los Angeles, California, USA; ^2^ Center for Bioinformatics and Functional Genomics, Samuel Oschin Comprehensive Cancer Institute, Cedars-Sinai Medical Center, Los Angeles, California, USA; ^3^ Women’s Cancer Program at the Samuel Oschin Comprehensive Cancer Institute, Cedars-Sinai Medical Center, Los Angeles, California, USA; ^4^ Departments of Medicine and Pathology, University of Southern California/Keck School of Medicine, Los Angeles, California, USA; ^5^ Department of Biomedical Science and Public Health, University of Derby, Derby, UK

**Keywords:** PAX8, transcription factor, ovarian cancer, chromatin immunoprecipitation, ChIP-seq

## Abstract

PAX8 is a lineage-restricted transcription factor that is expressed in epithelial ovarian cancer (EOC) precursor tissues, and in the major EOC histotypes. Frequent overexpression of PAX8 in primary EOCs suggests this factor functions as an oncogene during tumorigenesis, however, the biological role of PAX8 in EOC development is poorly understood. We found that stable knockdown of PAX8 in EOC models significantly reduced cell proliferation and anchorage dependent growth *in vitro,* and attenuated tumorigenicity *in vivo*. Chromatin immunoprecipitation followed by next generation sequencing (ChIP-seq) and transcriptional profiling were used to create genome-wide maps of PAX8 binding and putative target genes. PAX8 binding sites were significantly enriched in promoter regions (*p* < 0.05) and superenhancers (*p* < 0.05). MEME-ChIP analysis revealed that PAX8 binding sites overlapping superenhancers or enhancers, but not promoters, were enriched for JUND/B and ARNT/AHR motifs. Integrating PAX8 ChIP-seq and gene expression data identified PAX8 target genes through their associations within shared topological association domains. Across two EOC models we identified 62 direct regulatory targets based on PAX8 binding in promoters and 1,330 putative enhancer regulatory targets*. SEPW1,* which is involved in oxidation-reduction, was identified as a PAX8 target gene in both cell line models. While the PAX8 cistrome exhibits a high degree of cell-type specificity, analyses of PAX8 target genes and putative cofactors identified common molecular targets and partners as candidate therapeutic targets for EOC.

## INTRODUCTION

*PAX8* encodes a member of the paired box family of transcription factors (TFs) that is essential for normal embryonic development of the Müllerian ducts [1]. The role of PAX8 in the functioning of the kidney and thyroid is established; but more recently this TF been recognized as a key driver in the development of epithelial ovarian cancer (EOC). PAX8 is overexpressed in about 80% of the most common EOC histotype, high-grade serous ovarian cancer (HGSOC) [2-5]. PAX8 expression is routinely used clinically as a molecular maker to identify tumors of Müllerian origin [6, 7]. Different EOC histotypes arise from different cellular origins, many of which also express PAX8: normal fallopian tube secretory epithelial cells and ovarian surface epithelial cells, possible precursors of HGSOCs, and endometrial and endocervical epithelial cells, precursors of clear cell and endometrioid EOCs, all express PAX8 [7-9].

PAX8 may also be be involved in genetic susceptibility to ovarian cancer, possibly with subtype-specific biological roles in EOC initiation. The genomic region containing PAX8 is a common low penetrance susceptibility locus for mucinous ovarian cancer [10], and we recently identified PAX8 as a master regulator associated with susceptibility to serous ovarian cancer [11], suggesting that PAX8 may be a regulator of early EOC development and that transcriptional programs induced by PAX8 overexpression promote malignant transformation.

Although PAX8 is a common marker in HGSOC, the biological role of this gene is poorly understood in this subtype, and even less so in the other, less common histological subtypes. PAX8 knockdown in ovarian cancer cell lines reduces *in vitro* and *in vivo* tumorigenicity and induces apoptosis [12-15]. However, the functional role of PAX8 in EOC development is poorly understood. A detailed mechanistic understanding of the PAX8 cistrome will be key to any future development of agents targeting PAX8 as a therapy for EOC.

## RESULTS

### PAX8 expression in ovarian cancer histotypes

**Figure 1:**
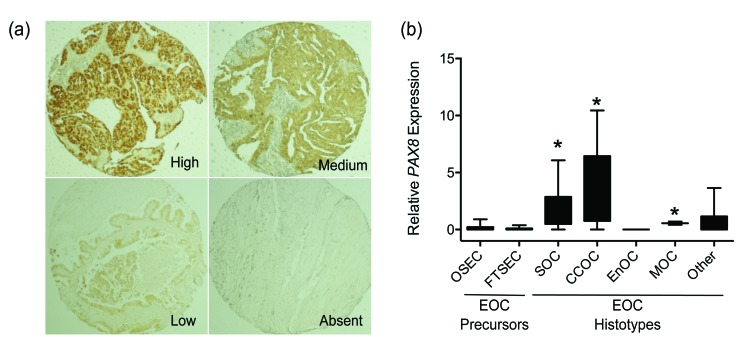
PAX8 gene expression in EOC subtypes and precursor cells **a.** Representative immunohistochemical staining for PAX8 in tumor specimens. **b.** PAX8 gene expression measured by qPCR. OSEC, ovarian surface epithelial cells; FTSEC, fallopian tube secretory epithelial cells; SOC, serous ovarian cancer; CCOC, clear cell ovarian cancer; EnOC, endometrioid ovarian cancer; MOC, mucinous ovarian cancer; EOC, epithelial ovarian cancer. Samples were relative to OSEC250, which has an average level of *PAX8* expression among OSECs, * *p* < 0.05 compared to OSECs and FTSECs, two-tailed unpaired T-test.

Analysis of The Cancer Genome Atlas (TCGA) data indicates that *PAX8* mRNA expression is significantly upregulated in high-grade serous ovarian cancers (HGSOC) compared to normal fallopian tube tissues (*p* = 0.007) (Table 1); but the role of PAX8 in other ovarian cancer histotypes is poorly understood. We used immunohistochemistry to evaluate PAX8 protein expression in ∼160 primary ovarian tumors representing the four major histotypes of EOC - HGSOC, clear cell ovarian cancer (CCOC), endometrioid ovarian cancer (EnOC) and mucinous ovarian cancer (MOC). Representative staining can be found in Figure 1a. PAX8 was most highly expressed in HGSOCs (88%) and CCOCs (89%) (*p* = 0.007), but was also expressed in over half of MOCs and EnOCs suggesting that PAX8 is a pan-EOC marker (Table 2). Highest PAX8 expression was associated with advanced stage carcinomas, compared to low stage carcinomas (*p* = 0.013), but did not correlate with tumor grade (Table 2).

**Table 1:** PAX gene expression in normal fallopian tubes and high-grade serous ovarian cancers from The Cancer Genome Atlas

Gene Name	P-value	Mean Expression in HGSOC (n=489)	Mean Expression in Normal FT (n=8)	Mean Difference HGSOC v FT
*PAX8*	0.007	0.044	-0.496	0.540
*PAX6*	1.22E-09	0.033	-0.435	0.468
*PAX7*	0.309	-0.016	0.018	-0.035
*PAX4*	0.056	-0.005	0.098	-0.103
*PAX1*	3.26E-05	-0.009	0.111	-0.120
*PAX9*	0.148	0.006	0.129	-0.123
*PAX3*	0.015	-0.020	0.193	-0.213
*PAX5*	0.041	-0.010	0.222	-0.231
*PAX2*	3.80E-06	-0.028	1.157	-1.184

Ranked in order of mean change in expression in tumors compared to normal tissues.

**Table 2:** PAX8 expression in EOC histotypes

	PAX8 staining N (%)		*P*-value^†^
Clinical Feature	Low/Absent	Medium/High	
Stage			
1	17 (30)	39 (70)	0.013*
2	6 (21)	22 (79)	
3	16 (14)*	101 (86)*	
4	5 (20)	20 (80)	
			
Grade			
G1	2 (7)	25 (93)	0.072
G2	11 (24)	35 (76)	
G3	30 (20)	121 (80)	
			
Residual Disease			
Optimal debulking	21 (22)	73 (78)	0.391
Sub-optimal debulking	22 (17)	106 (83)	
			
Histological type			
Serous	15 (12)*	109 (88)*	0.007*
Mucinous	6 (35)	11 (65)	
Endometrioid	9 (29)	22 (71)	
Clear Cell	2 (11)*	16 (89)*	
Undifferentiated	10 (29)	24 (71)	
Borderline	3 (43)	4 (57)	

A significantly higher number of stage 3 and serous and clear cell ovarian carcinomas express high/medium levels of PAX8 (denoted by *). ^†^The Pearson Chi Squared test was used to derive the *p*-values.

We also performed semi-quantitative real-time RT-PCR (RT-qPCR) in 72 primary ovarian cancer precursor cells (66 normal ovarian epithelial and 6 normal fallopian tube secretory epithelial cell cultures) and 58 ovarian cancer cell lines derived from a range of ovarian cancer histotypes. These data were consistent with the results of immunohistochemistry analyses: *PAX8* was expressed at higher levels in EOC cell lines compared to normal precursor cells (*p* < 0.0001, two-tailed unpaired T-test). Highest *PAX8* expression was observed in HGSOC and CCOC cell lines (Figure 1). There was significant variation in *PAX8* expression across EOC histotypes (*p* < 0.0002, one-way ANOVA), with *PAX8* overexpressed in serous ovarian cancer (n=16), CCOC (n=18) and MOC cell lines (n=2), but not in EnOC lines (n=3) lines. Taken together, these data suggest that PAX8 may function as an oncogene in the development of most EOC histotypes.

### PAX8 knockdown reduces anchorage dependent and independent growth

**Figure 2:**
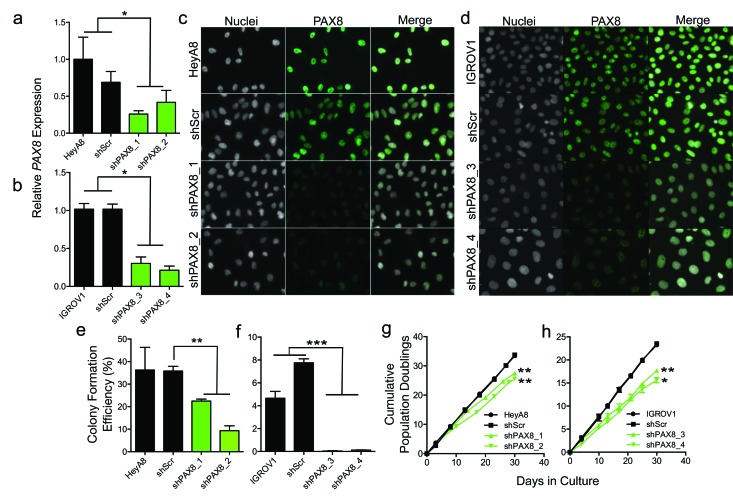
I*n vitro* analysis of PAX8 knockdown models PAX8 was stably knocked down using short hairpin RNAs (shRNAs). *PAX8* gene expression in knockdown and control lines in **a.** HeyA8 and **b.** IGROV1 ovarian cancer cell lines. Knockdown of PAX8 protein was confirmed by immunofluorescent staining in **c.** HeyA8 and **d.** IGROV1 cells. Nuclei were counterstained with Hoescht DNA stain. 200X magnification is shown. **e.**-**f.** Anchorage independent growth assays in (**e**) HeyA8 and (**f**) IGROV1 PAX8 knockdown models. (**g-h**) Anchorage dependent growth assays in **g.** HeyA8 and **h.** IGROV1 models. Data shown are mean ± standard deviation, and are representative of at least three independent experiments. * *p* < 0.05, ** *p* < 0.01, *** *p* < 0.001, two-tailed paired T-test. In panels (**g**) and (**h**) T-tests values (two tailed, paired) for knockdown lines compared to shScr lines are indicated.

*PAX8* was stably knocked down using two independent shRNAs, in the HeyA8 and IGROV1 EOC cell lines. Both lines represent models of high-grade ovarian adenocarcinoma but no specific histological subtype. A control line expressing a non-silencing ‘scrambled’ shRNA hairpin (shScr) was also generated for each line. In HeyA8 we achieved a 74% and 58% reduction in *PAX8* expression for shPAX8_1 and shPAX8_2 transduced lines respectively (*p* > 0.05) (Figure 2a). In IGROV1 we achieved 70% and 79% reduction in *PAX8* expression for shPAX8_3 and shPAX8_4, respectively (*p* > 0.05) (Figure 2b). In both cell lines we observed negligible changes in *PAX8* expression in shScr controls compared to parental cells. Immunofluorescent staining confirmed a marked reduction in PAX8 protein expression in shPAX8 transduced cell lines compared to shScr and parental controls (Figure 2c & 2d).

PAX8 knockdown was associated with a significant reduction in anchorage-independent growth compared to shScr (HeyA8 shPAX8_1, *p* = 0.003; HeyA8 shPAX8_2, *p* = 0.003; IGROV1 shPAX8_3, *p* = 0.0008; IGROV1 shPAX8_4, *p* = 0.0007) (Figure 2e & 2f). We also observed significant increases in population doubling times in all PAX8 knockdown models compared to shScr cell lines (HeyA8 shPAX8_1, *p* = 0.004; HeyA8 shPAX8_2 *p* = 0.004; IGROV1 shPAX8_3, *p* = 0.008; IGROV1 shPAX8_4, *p* = 0.011) (Figure 2g & 2h).

### PAX8 knockdown impairs tumorigenicity *in vivo*

**Figure 3:**
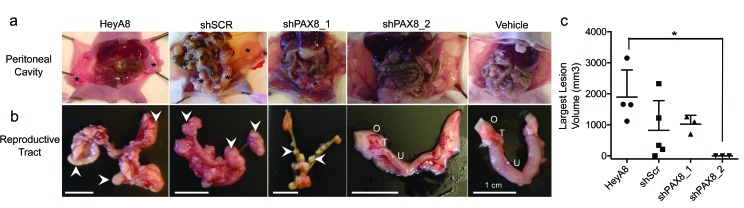
*In vivo* analysis of PAX8 knockdown models **a.** *In vivo* growth of HeyA8 PAX8 models, representative images of peritoneal cavities. Tumors are indicated with an asterisk. **b.** Reproductive tracts in xenograft models, tumors are indicated with arrowheads. O, ovary; T, oviduct (murine fallopian tube); U, uterine horn; shScr, non-targeting control shRNA. **c.** Quantitative analysis of largest tumor volume. * *p* < 0.05 *versus* parental cells. Two tailed unpaired T-test.

We next examined the effects of PAX8 knockdown on *in vivo* tumorigenicity in HeyA8 models. For parental and shScr-expressing HeyA8 cells we observed abdominal distention four weeks after intraperitoneal injection of tumor cells, indicative of significant tumor burden (Figure 3a & 3b). Detailed examination of the abdominal cavity during necropsy identified tumor spread throughout the peritoneal cavity in all 4 HeyA8 and in 4/5 HeyA8+shScr injected mice. Pathological examination of tumors formed in HeyA8 and HeyA8+shScr mice classified these tumors as high-grade poorly differentiated adenocarcinomas, consistent with the known pathology of the HeyA8 cancer cell line. In contrast, HeyA8+shPAX8-1 injected mice demonstrated modest tumor growth, with smaller tumors compared to controls. There was no evidence of tumor growth in HeyA8+shPAX8_2 mice (n=3) (Figure 3c). Subsequent pathological analyses of HeyA8+shPAX8_2 mice confirmed the absence of tumor deposits in peritoneal tissues harvested from these mice, which resembled the phenotype of mice injected with vehicle alone (n=3).

### Characterizing the PAX8 cistrome

To characterize the PAX8 cistrome we performed chromatin immunoprecipitation followed by next generation sequencing (ChIP-seq) to catalogue PAX8 binding sites in HeyA8 and IGROV1 cell lines. In parallel we performed ChIP-seq for acetylation of lysine 27 on histone subunit 3 (H3K27ac) to identify when PAX8 binding coincided with active chromatin.

**Figure 4:**
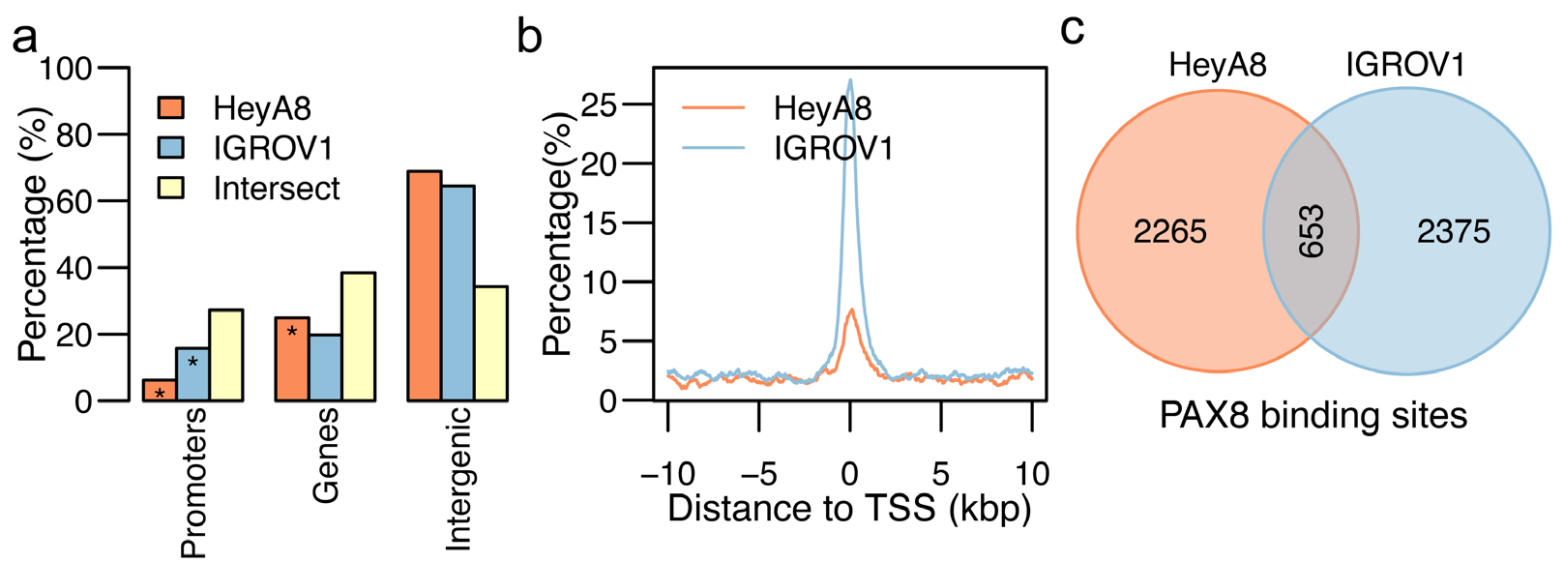
PAX8 ChIP-seq binding sites for HeyA8 and IGROV1 **a.** Distribution of PAX8 binding sites across promoter, gene body, superenhancer, and enhancer regions of the human genome for PAX8 binding sites in HeyA8 (orange), IGROV1 (blue), and the intersection of the two sets (yellow). Distribution of the distance of the PAX8 binding sites in HeyA8 (orange) and IGROV1 (blue) with regards to the closest transcription start site (TSS) normalized by the number of PAX8 binding sites in HeyA8 and IGROV1, respectively. Venn diagrams showing the number of (**c**) PAX8 binding sites in HeyA8 (orange) and IGROV1 (blue).

We identified 2918 sites (average length 566 bp) throughout the genome where PAX8 binds in HeyA8 cells, and 3028 PAX8 binding sites (average length = 673 bp) in IGROV1 cells. PAX8 binding sites were significantly enriched in promoter regions in both cell lines (*p* < 0.05, Fisher’s exact test). Approximately 6% (n=182) and 16% (n=478) of all PAX8 binding sites fell in promoter regions in HeyA8 and IGROV1 cells, respectively (Figure 4a & 4b). Although more than 60% of PAX8 binding sites were outside promoters or gene bodies, we did not find evidence for enrichment in intergenic regions, even when repeat-rich regions were excluded. There were 653 PAX8 binding sites in common between the two cell lines (Figure 4c). There were some significant differences in the PAX8 cistrome between the two cell lines: for example, 60% (n=1829) of PAX8 binding sites in IGROV1 overlapped with H3K27ac positive regions compared to only ∼25% (n=731) of PAX8 binding sites in HeyA8 cells, even though we observed 2.5 times more H3K27ac positive regions in HeyA8 cells.

We catalogued superenhancers in the two cell lines, defined as enhancers >10kb that are highly enriched in H3K27ac and lineage-specific transcription factors [16]. Based on the H3K27ac ChIP-seq data we characterized 391 superenhancers (average length = 126K bp) in HeyA8 and 523 superenhancers (average length = 71K bp) in IGROV1. HeyA8 PAX8-binding sites were significantly enriched at superenhancers; there were approximately 326 (11%) PAX8 binding sites in superenhancer regions (odds ratio 1.82; *p* < 0.01).

### Characterizing the PAX8 binding motif

**Figure 5:**
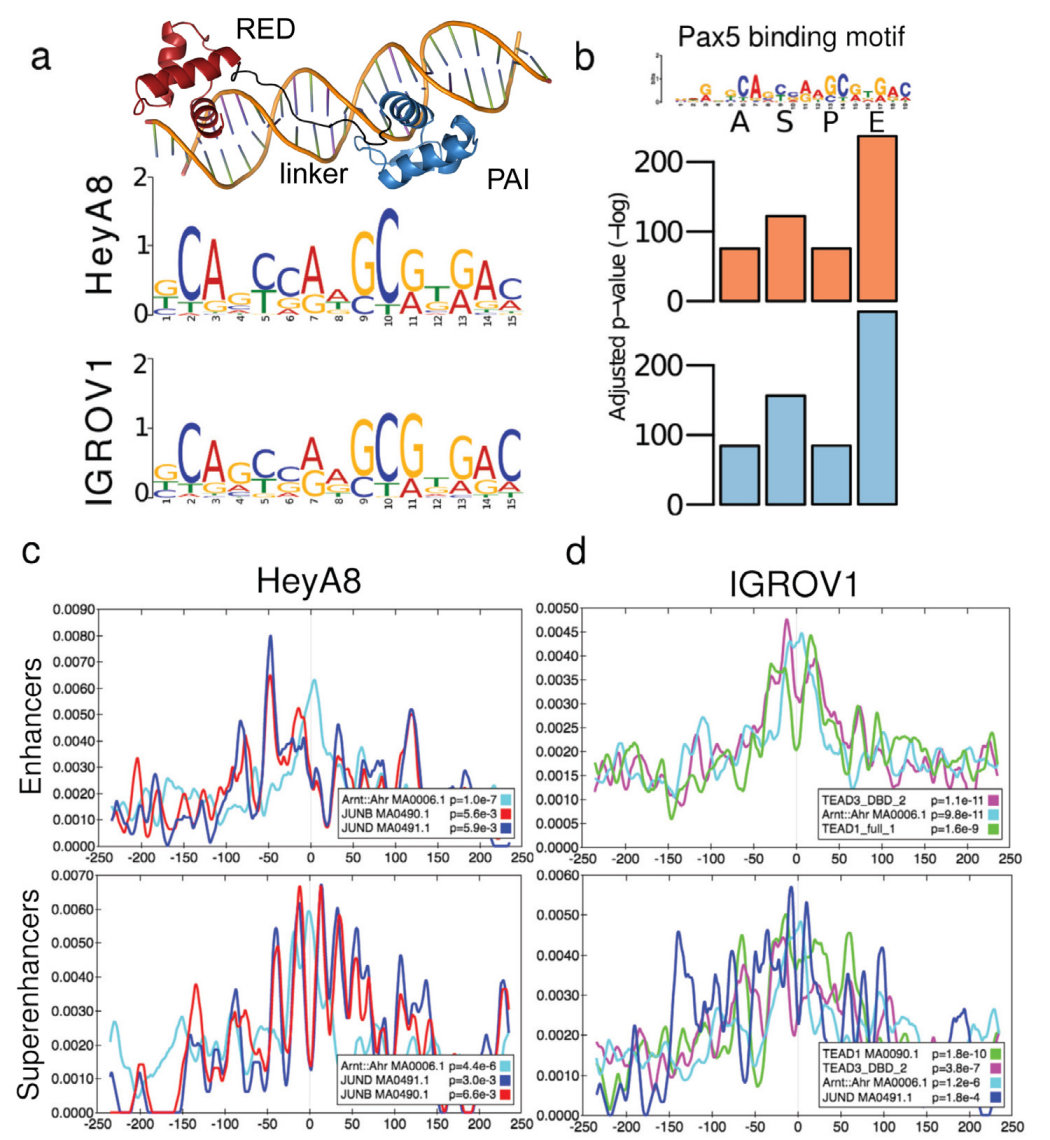
Defining the PAX8 binding motif and identifying candidate co-regulators **a.** A model of the DNA binding domain of PAX8. Binding motifs identified by MEME-ChIP using the H3K27ac positive PAX8 binding sites in the enhancer set for HeyA8 and IGROV1. **b.** Pax5 binding motif (from Jaspar [43]) was selected as the closest binding motif to the primary PAX8 binding motif based on the adjusted p-value across four sets of PAX8 binding sites used for motif discovery (all (A), superenhancers (S), promoters (P), and enhancer (E) PAX8 peaks) for HeyA8 (orange) and IGROV1 (blue). Significantly enriched motifs within ±250 bp of the summits of PAX8 peaks in **c.** HeyA8 and (d) IGROV1 cells. Other than PAX-like motifs, only motifs identified in both cell lines are shown (with the exception of TEAD1/3, which was unique to IGROV1). The grey line at 0 bp indicates the summit of the PAX8 peak.

We used MEME-ChIP suite to characterize a PAX8 binding motif from the PAX8 ChIP-seq data from each cell line. For HeyA8, we included 2,923 sequences centered in the summit of the PAX8 binding sites. The motif shown in Figure 5a for HeyA8 was present in 2,127 (73%) of all the sequences, and centered, on average, 4.5 bp off the center of the sequences. For IGROV1, we used 3,029 total input sequences centered in the summit of the IGROV1 PAX8 binding sites, from which 1,415 (47%) have the motif shown in Figure 5a for IGROV1, and the motif is centered, in average, 1.5 bp off the center of the input sequences. Crucially, we identified the same PAX-like motif in both cell lines. This motif correlates with a PAX8-dsDNA homology model we generated using the PAX5-dsDNA complex (PDB id: 1k78, sequence identity = 85%) as a template. The homology model has high structure similarity to this template (RMSD = 0.2) and to another PAX8 homolog, PAX6-dsDNA (PDB id: 6pax; sequence identity = 72%; RMSD = 1.6). The protein side-chain/DNA-base contacts observed in the homology model extend 17 base pairs. First, the PAI subdomain makes contact with 5 continuous bases in the major groove and one base in the minor groove, followed by the linker that makes contact with 3 non-continuous bases in the minor groove. At the C terminus of the PAIRED DNA binding domain, the RED subdomain makes contact with 4 continuous bases via the major groove. Since it is known that protein side-chain/DNA-major groove contacts provide higher specificity than contacts in the minor groove, the expected motif would have a region of medium to low specificity (provided by the linker) in the middle, flanked by a couple of highly-specific bases (provided by the PAI and RED subdomains), exactly the pattern exhibited in the PAX-like motif we identified (Figure 5a).

### Identifying PAX8 candidate co-regulators

To identify candidate co-regulators, that may cooperate with PAX8 to regulate gene expression, we divided all PAX8 peaks that overlap H3K27ac into three non-overlapping sets: (1) PAX8 binding sites that overlap superenhancer regions, (2) PAX8 binding sites that overlap promoter regions defined as 1000 bp upstream and 100 bp downstream of the transcription start site of a gene, and (3) PAX8 binding sites that neither overlap superenhancers, nor promoters, but overlap H3K27ac marks (i.e. typical enhancers). PAX motifs were significantly enriched in all classes of PAX8 binding sites in both cell lines (Figure 5b, Table 3). In both cell lines we found enrichment of Arnt::Ahr motifs for PAX8 binding sites overlapping superenhancers or enhancers, but not promoters (in HeyA8 superenhancers, *p* = 4.37x10^-6^; in HeyA8 typical enhancers, *p* = 9.98x10^-8^; in IGROV1 superenhancers, *p* = 1.19x10^-6^; in IGROV1 typical enhancers *p* = 9.76x10^-11^) (Figure 5c & 5d, Table 3). In addition, JUND and JUNB motifs were enriched in superenhancers in both cell lines (in HeyA8 JUND, *p* = 3.00x10^-3^; JUNB, *p* = 6.60x10^-3^; in IGROV1, JUND *p* = 1.82x10^-4^; JUNB, *p* = 2.75x10^-4^). A recent report identified TEAD as an important PAX8 co-regulator [17]. In our dataset a TEAD-like motif flanked the PAX-like motif in IGROV1 but not in HeyA8 (TEAD3 enrichment in IGROV1 superenhancers, *p* = 1.76x10^-10^, in IGROV1 typical enhancers, *p* = 9.46x10^-6^; TEAD1 enrichment in IGROV1 superenhancers, *p* = 3.85x10^-7^, in IGROV1 typical enhancers, *p* = 1.07x10^-11^) (Figure 5c & 5d, Table 3).

**Table 3:** Candidate PAX8 co-regulators

Motif/Factor	HeyA8				IGROV1			
	All	Super enhancer	Promoter	Enhancer	All	Super enhancer	Promoter	Enhancer
PAX5	-75.64	-122.14	-75.64	-237.09	-84.48	-156.36	-84.48	-276.91
PAX2	-76.06	-140.02	-76.06	-242.56	-90.26	-202.7	-90.26	-253.45
PAX1	-42.29	-85.93	-42.29	-121.7	-38.96	-114.76	-38.96	-138.49
PAX6	-38.46	-121.77	-38.46	-133.79	-16.11	-94.49	-16.11	-127.34
PAX9	-35.48	-58.45	-35.48	-83.5	-26.75	-76.52	-26.75	-95.52
TEAD3	0	0	0	0	0	-14.77	0	-25.26
Arnt::Ahr	0	-12.34	0	-16.12	0	-13.64	0	-23.05
TEAD1	0	0	0	0	0	-22.46	0	-11.55
Zbtb3_primary	0	0	0	-7.92	0	0	0	-14.5
GMEB2	0	0	0	-6.17	0	0	0	-14.07
Klf4	0	0	0	0	0	0	0	-11.27
Zfp161_secondary	0	0	0	0	0	0	0	-9.9
JUND	0	-5.81	0	-5.13	0	-8.61	0	0
SP1	0	0	0	0	0	0	0	-8.61
Sp4_secondary	0	0	0	0	0	0	0	-8.25
FOS	0	-5.52	0	-5.23	0	0	0	-8.21
JUNB	0	-5.02	0	-5.18	0	-8.2	0	0
HIF1A::ARNT	0	0	0	0	0	-7.76	0	-6.05
Klf1	0	0	0	0	0	0	0	-7.47
KLF5	0	0	0	0	0	0	0	-6.28
Nfe2l2	0	0	0	-6.2	0	0	0	0
Max_secondary	0	0	0	0	0	0	0	-5.71
NFIA	0	0	0	0	0	0	0	-5.43
Klf7_primary	0	0	0	0	0	0	0	-5.19
KLF14	0	0	0	0	0	0	0	-5.12
NFYA	0	0	0	0	0	0	0	-5.07

Log adjusted p-values of motifs found within 50 bases of PAX8 binding site summits of HeyA8 and IGROV1 using all, superenhancer, promoter and enhancer-associated PAX8 binding sites.

### Identifying PAX8 target genes

Global gene expression analysis was performed to identify genes that are differentially expressed following PAX8 knockdown in HeyA8 and IGROV1 cell lines. Using a fold-change cutoff of 1.2, we identified 1,055 and 1,293 differentially expressed genes (DEGs) in HeyA8 and IGROV1 models respectively (FDR < 0.05; Supplementary Table 1, validation). Table 4 lists the DEGs from each cell line; Table 5 lists with the genes common to both cell lines. We integrated gene expression changes with PAX8 binding sites to map PAX8 binding sites to target genes, leveraging topological association domains (TADs) coordinates from human embryonic stem cells [18] to annotate DEGs. TADs are megabase-scale regions, largely stable across different cell types, of increased local interaction frequency [18]. Using these annotations, we divided associations with PAX8 binding sites into 3 categories: (1) direct regulatory targets, defined as DEGs that have a PAX8 binding site in their promoter region; (2) putative regulatory targets, defined as DEGs that have a PAX8 binding site within the same TAD but lacking PAX8 binding in the promoter; and (3) indirect regulatory targets, defined as DEGs where there is no PAX8 binding site within the same TAD or the promoter.

**Table 4:** Significantly changing genes in EOC models following PAX8 knockdown

HeyA8			IGROV1		
Gene Name	Fold Change	P-value	Gene Name	Fold Change	*P*-value
Downregulated Genes			Downregulated Genes		
*DCDC2*	-2.8	8.82E-07	*SPON1*	-13.4	3.50E-10
*SLC7A5*	-2.6	1.09E-03	*KRT24*	-12.0	4.32E-12
*CDH5*	-2.1	1.12E-05	*MMP7*	-6.8	4.77E-06
*FAM167A*	-2.1	1.25E-05	*APBB1IP*	-3.3	2.06E-07
*C8ORF13*	-2.0	2.25E-05	*LOC644612*	-3.3	5.00E-04
*H2AFY2*	-2.0	1.23E-03	*THY1*	-3.2	1.84E-08
*IL11*	-2.0	2.37E-04	*C1ORF85*	-3.1	8.82E-09
*C13ORF15*	-2.0	7.73E-09	*STK32B*	-3.0	2.16E-05
*PAX8*	-1.9	4.57E-06	*SAMD5*	-3.0	4.90E-06
*SCD*	-1.9	6.61E-06	*CYP4F11*	-2.8	5.45E-06
Upregulated Genes			Upregulated Genes		
*IL1B*	2.6	3.92E-05	*LOC149501*	8.0	1.19E-09
*LOC644350*	2.6	7.50E-07	*KRT18P13*	8.2	2.23E-10
*SCG5*	2.7	1.92E-06	*MGC42367*	8.4	8.26E-10
*STC1*	2.7	2.51E-05	*LOC399965*	8.8	6.92E-11
*HNRPLL*	2.7	5.59E-11	*EMP1*	9.1	3.39E-11
*IL8*	3.3	3.18E-05	*ANKRD1*	10.8	4.06E-09
*LPXN*	3.5	3.06E-07	*LOC644743*	11.0	1.35E-11
*IL13RA2*	3.9	4.50E-14	*KRT8*	12.2	1.74E-11
*LOC728285*	4.2	8.81E-09	*LOC647954*	12.2	5.67E-12
*TGFBI*	6.2	5.51E-09	*CDH17*	20.4	6.37E-10

**Table 5:** Genes commonly changing in HeyA8 and IGROV1 models following PAX8 knockdown

	HeyA8			IGROV1	
Gene Name	Fold Change	P-value	Fold Change		*P*-value
*AJAP1*	-1.9	3.82E-10	-1.8		2.17E-09
*ANKRD1*	1.6	2.02E-03	10.8		4.06E-09
*C20orf75*	-1.7	9.06E-05	-2.8		8.22E-05
*HIST1H2BK*	1.9	2.20E-05	1.8		1.33E-05
*HIST2H2AA3*	2.0	8.41E-07	1.7		2.24E-06
*HIST2H2AA4*	1.9	1.79E-06	1.6		8.88E-07
*HIST2H2AC*	1.6	1.35E-06	1.5		3.62E-04
*PAX8*	-1.9	4.57E-06	-2.3		6.51E-08
*PRSS23*	1.5	2.10E-04	3.9		6.86E-10
*SAT1*	1.8	4.47E-07	1.7		2.75E-05
*SIRPA*	-1.7	2.56E-05	-1.7		1.48E-07
*STC1*	2.7	2.51E-05	1.8		2.18E-05
*TGFBR2*	1.8	1.46E-05	1.5		2.41E-05
*VASN*	1.7	4.02E-07	6.0		5.38E-11

**Figure 6:**
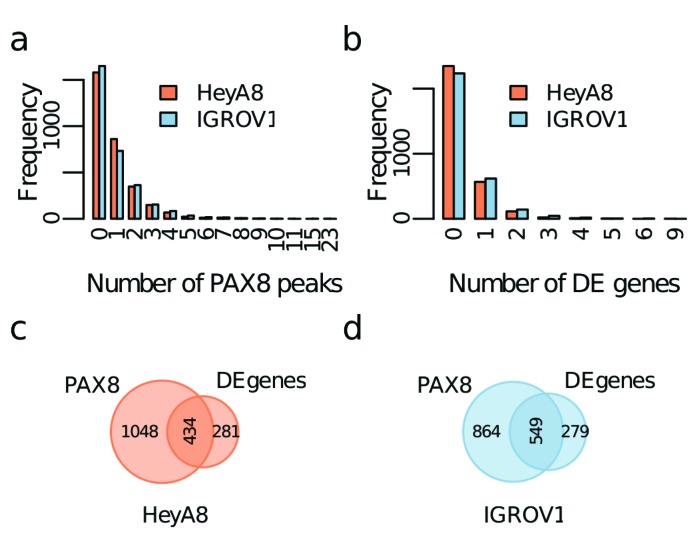
Identification of PAX8 regulatory targets using topological association domains (TADs) **a.** Distribution of the number of PAX8 peaks per TAD. **b.** Distribution of the number of differentially expressed genes (DEGs) per TAD. Venn diagrams showing the number of TADs with at least one PAX8 peak or at least one DEG for **c.** HeyA8 and **d.** IGROV1.

There were 3,062 TADs with an average length of 852.2 Kbp (range 80 Kbp - 4.44 Mbp), of which 1,482 in HeyA8 and 1,413 in IGROV1 contained PAX8 binding sites. About two thirds of TADs (2,012) had a PAX8 peak in at least one of the cell lines, with 884 (29%) shared between HeyA8 and IGROV1 models. More than half of the TADs containing PAX8 binding sites - 862/1483 (58%) in HeyA8 and 732/1413 (52%) in IGROV1 - contained just one or two PAX8 binding sites (Figure 6a). We found no correlation between the length of the TAD and the number of PAX8 binding sites. About a quarter of TADs - 714 TADs in HeyA8 and 830 in IGROV1 - contained PAX8 regulated DEGs and most TADs harboring DEGs (566 and 619 for HeyA8 and IGROV1 respectively) contained a single DEG (Figure 6b). The maximum number of DEGs identified within the same TAD was 9 in HeyA8 and 6 in IGROV1; there was no correlation between the length of the TAD and the number of DEGs. There were 434 and 549 TADs containing at least one PAX8 binding site *and* one DEG in HeyA8 and IGROV1 cell models respectively (Figure 6c-6d). The mean distance in base pairs to the nearest DEG within the same TAD was 309 Kbp (sd=357 Kbp) in HeyA8 and 235 Kbp (sd=275 Kbp) in IGROV1.

From the 1,055 DEGs for HeyA8, we identified 16 direct regulatory targets based on PAX8 binding in the promoter, and 575 putative enhancer regulatory targets based on the presence of a PAX8 binding site within the same TAD. From the 1,293 DEGs for IGROV1, we identified 46 direct regulatory targets and 755 as putative enhancer regulatory targets. The larger number of direct regulatory targets identified in IGROV1 compared to HeyA8 reflects the higher global enrichment of PAX8 binding sites in promoter regions in this cell line. We identified *SEPW1* as a direct regulatory target in both cell lines and 54 additional genes that are both differentially expressed following PAX8 knockdown *and* have PAX8 binding at an enhancer in the same TAD in both cell lines (Table 6).

**Table 6:** PAX8 putative enhancer regulatory targets

Gene Symbol	HeyA8		IGROV1	
	p-value	Fold change	*p*-value	Fold change
*ANKRD1*	2.02E-03	1.57	4.06E-09	10.79
*VASN*	4.02E-07	1.74	5.38E-11	6.04
*IL1B*	3.92E-05	2.57	1.10E-04	1.26
*PAX8*	4.57E-06	-1.95	6.51E-08	-2.35
*CLIC3*	1.19E-04	1.25	1.58E-03	2.07
*HIST2H2AA3*	8.41E-07	2.00	2.24E-06	1.73
*AJAP1*	3.82E-10	-1.94	2.17E-09	-1.77
*SLC26A2*	1.50E-03	-1.29	2.51E-06	-1.91
*HIST1H2BK*	2.20E-05	1.90	1.33E-05	1.85
*GCLM*	5.78E-06	-1.33	3.94E-05	-1.87
*HIST2H2AA4*	1.79E-06	1.86	8.88E-07	1.56
*MXD4*	3.62E-04	1.73	1.10E-04	1.35
*ELFN2*	7.37E-05	-1.50	7.67E-06	-1.72
*RRAS2*	6.42E-04	-1.45	5.91E-08	-1.69
*CTSL2*	1.65E-04	-1.41	6.56E-09	-1.61
*SLC25A15*	6.35E-07	-1.59	4.85E-06	-1.61
*HIST2H2AC*	1.35E-06	1.59	3.62E-04	1.50
*ATOX1*	6.52E-05	1.26	8.64E-07	1.52
*CASP1*	3.96E-08	1.52	5.99E-04	1.24
*S100A13*	7.89E-06	-1.51	2.82E-04	-1.36
*EVI5L*	8.13E-06	1.26	9.11E-07	1.46
*CEP55*	5.11E-05	-1.45	2.16E-03	-1.21
*UBA7*	7.76E-05	1.44	7.35E-06	1.29
*CHAF1A*	5.70E-05	-1.24	1.21E-04	-1.43
*SKA3*	9.17E-04	-1.36	7.51E-04	-1.41
*SH3BGRL3*	5.28E-08	1.41	3.79E-04	1.23
*PTPRF*	8.77E-10	1.41	1.32E-05	1.35
*S100A6*	5.02E-04	1.34	4.37E-05	1.41
*NRP1*	4.26E-06	1.36	1.09E-05	1.39
*EHD2*	4.79E-04	1.29	6.48E-04	1.39
*XPO4*	2.85E-03	-1.36	2.78E-05	-1.37
*RHOC*	7.17E-07	1.37	1.26E-04	1.24
*KIAA1539*	2.13E-07	1.29	4.36E-04	1.36
*JARID2*	1.27E-03	1.28	8.05E-06	1.36
*CASP4*	2.59E-03	1.21	3.46E-05	1.36
*TROAP*	1.02E-03	-1.31	2.81E-03	-1.36
*DUSP28*	1.25E-03	1.31	7.97E-05	1.34
*SEPW1*	6.62E-05	1.33	1.63E-03	1.30
*FEN1*	9.88E-06	-1.33	8.67E-05	-1.23
*ACAA2*	1.45E-03	-1.21	5.36E-05	-1.33
*MPHOSPH8*	7.94E-04	-1.30	4.22E-04	-1.31
*POLR3K*	4.38E-04	-1.27	1.83E-04	-1.31
*FOXM1*	2.30E-06	-1.29	1.24E-04	-1.26
*NUP210*	2.99E-03	-1.20	9.35E-05	-1.29
*POLD1*	7.56E-08	-1.28	1.92E-03	-1.23
*CHTF18*	3.49E-04	-1.26	2.28E-03	-1.28
*CDCA5*	1.41E-03	-1.24	6.80E-05	-1.28
*DSCC1*	2.08E-05	-1.28	4.92E-05	-1.28
*FRAT2*	8.96E-04	-1.26	1.82E-03	-1.20
*GPRIN1*	1.16E-04	-1.25	3.79E-04	-1.25
*ZNF259*	3.22E-04	-1.24	7.71E-05	-1.25
*FANCG*	1.99E-03	-1.21	1.79E-04	-1.25
*OIP5*	1.46E-04	-1.22	2.65E-03	-1.25
*M6PRBP1*	1.99E-05	1.22	1.95E-03	1.20

Shown are the DEGs changing in the same direction when PAX8 is knocked down in HeyA8 and IGROV1, sorted by absolute fold change.

**Figure 7:**
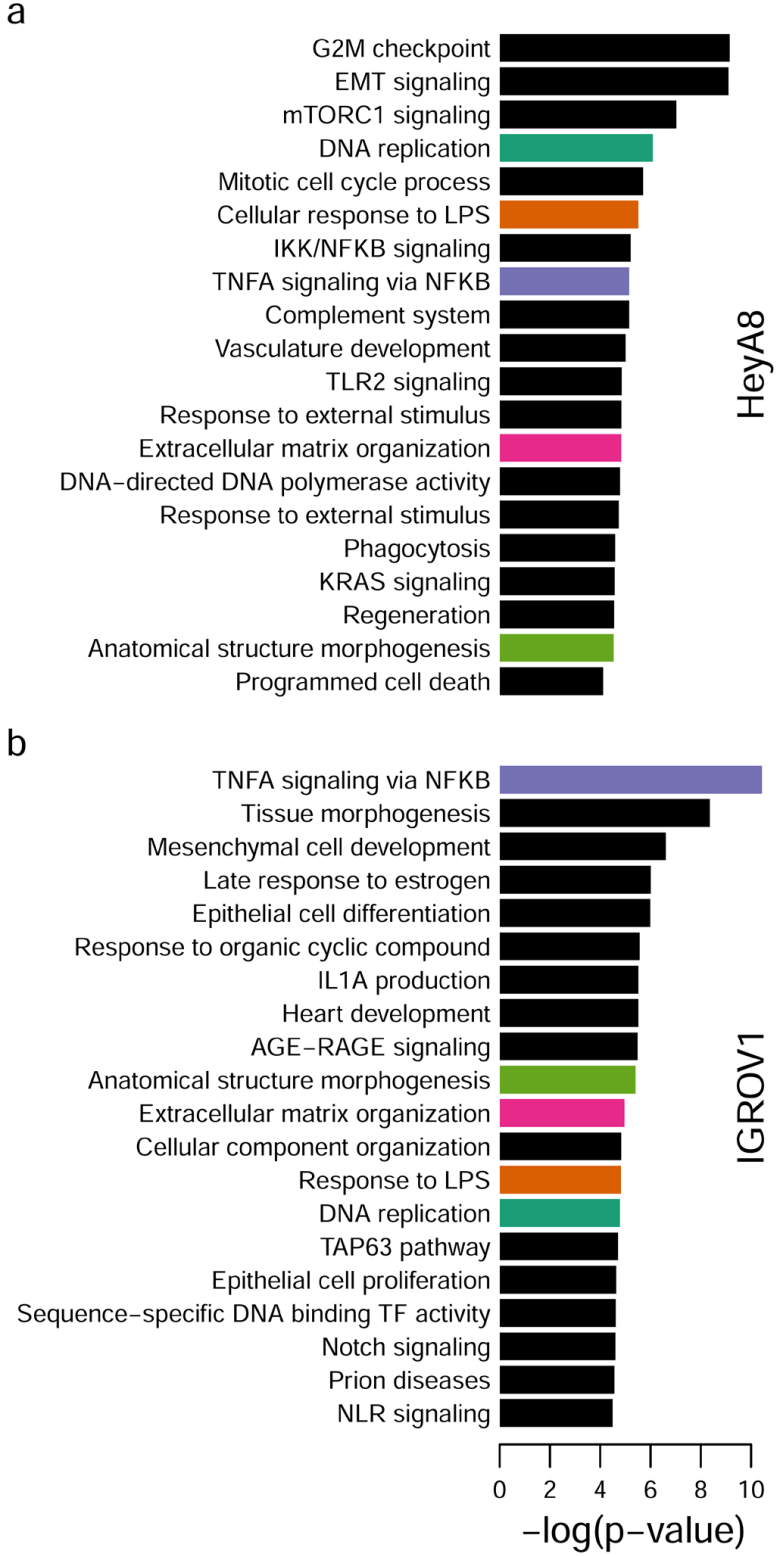
Pathway enrichment analysis **a.** Top 20 pathways and gene sets enriched using 9 direct and 462 putative enhancer regulatory targets for PAX8 in HeyA8. **b.** Top 20 pathways and gene sets enriched using 46 direct and 739 putative enhancer PAX8 regulatory targets in IGROV1. DNA replication (green), cellular responses to LPS (orange), TNFA signaling (purple) extracellular matrix organization (pink) and morphogenesis (light green) overlap between HeyA8 and IGROV1 top 20 enriched pathways.

Finally, we used both direct and putative enhancer regulatory targets for HeyA8 (589 DEGs) and IGROV1 (801 DEGs) to perform pathway enrichment analysis using Metascape [19]. We identified 5 enriched pathways in common in both HeyA8 and IGROV1 cell lines: DNA replication, response to lipopolysaccharide (LPS), tumor necrosis factor alpha (TNFA) signaling via nuclear factor-kappaB (NFKB), extracellular matrix organization and anatomical structure morphogenesis. The top 20 enriched gene sets and pathways are shown in Figure 7.

## DISCUSSION

Characterizing transcription factors (TFs) deregulated during tumorigenesis has provided key insights into disease etiology, disease origins, and therapeutic targeting for many tumor types. By identifying the TFs responsible for higher-order deregulation of gene expression we can better understand the evolution of the key transcriptional networks driving tumorigenesis and more potently target tumor cells by inhibiting a factor that simultaneously deregulates tens to hundreds of proto-oncogenes and tumor suppressor genes. Examples of such factors include the androgen receptor (AR), a TF involved in normal prostate development which becomes reprogrammed during prostate carcinogenesis [20]. AR is targeted by anti-androgenic therapies used for the treatment of prostate cancer and in tumors cooperates with HOXB13 [20], a gene that when mutated causes predisposition to prostate cancer [21]. Similarly, the estrogen receptor (ER) is targeted by tamoxifen for the treatment of ER positive breast cancer, and co-operates with the FOXA1 TF to drive breast cancer progression [22].

The role of TFs deregulated in epithelial ovarian cancer (EOC) are poorly characterized, although a handful of TFs and chromatin remodelers are known to be deregulated in a histotype-specific manner. For example, WT1 is a marker of serous tumors, and *ARID1A* is somatically mutated in ∼50% of clear cell and ∼30% of endometrioid tumors [23]. In this study we sought to understand in detail the mechanistic underlying the putative master regulator of EOC development, PAX8. PAX8 has most frequently been associated with the development of high-grade serous ovarian cancers (HGSOCs); but as this and other studies have shown, PAX8 is commonly expressed by other major EOC histotypes. Our phenotypic characterization in PAX8 knockdown models is consistent with previous studies showing that PAX8 is involved in proliferation of EOC cells *in vitro*, and PAX8 knockdown impairs tumor formation *in vivo* [12-15].

We sought to characterize, in detail, the PAX8 cistrome, as the binding regions for this factor have only been delineated for a small number of cell lines, and only in HGSOC [24]. To characterize the landscape of PAX8 binding within the context of chromatin state, we integrated PAX8 ChIP-seq data with maps of global histone activation generated by performing ChIP-seq for H3K27ac in the same two EOC cell lines. To link PAX8 binding sites to target genes, we measured differential gene expression following PAX8 knockdown. Many studies assign TF ChIP-seq peaks to DEGs within a specified genomic window to map associations between DEGs and TF binding sites. However, the selection of the window size can often be arbitrary leading to results that might not be robust. Since the genome is subdivided into topological association domains (TADs) that represent regions of high interaction frequencies, we instead leveraged TAD boundaries to link PAX8 binding sites to PAX8 target genes. In this study, we report that while PAX8 can regulate target gene expression *via* promoter binding, most PAX8 binding occurs outside of promoter regions, confirming a previous observation [24].

We explored non-promoter PAX8 binding sites in more detail using the H3K27ac ChIP-seq data and found that PAX8 binding was enriched at superenhancers, regions of dense H3K27ac signal that are commonly found at genes involved in differentiation, development and tumorigenesis [24]. A lack of enriched motifs for other co-factors suggests that PAX8 may function as a solo TF at promoters; alternatively, PAX8 may be a promiscuous TF at promoters, interacting with many other co-factors in a gene-specific manner. However, at enhancers and superenhancers, PAX8 binding sites preferentially co-occurred within close proximity of the motifs of a select handful of TFs. Of particular interest are the Arnt::Ahr and JUND/JUNB motifs. JUND/JUNB, as well as TEAD1/TEAD3 motifs (in the IGROV1 cell line) flank the summit of PAX8 ChIP-seq peaks, which may indicate co-binding of these two factors to adjacent DNA. The Arnt::Ahr motif is enriched at the summit of the PAX8 ChIP-seq peak, suggesting an alternative mechanism of co-regulation. Functional assays will be required to verify cooperation between PAX8 and these novel cofactors. Even so, there are potentially significant translational implications of these findings. Should an anti-PAX8 therapy be developed, a combination therapy targeting both PAX8 and its preferred cofactors may confer the most potent effects. Even in the absence of a PAX8 inhibitor, inhibiting these cofactors may be sufficient to inactivate oncogenic PAX8 signaling and impair tumor growth. Functionally, these are promising co-targets. The aryl-hydrocarbon receptor (AHR) nuclear translocator (ARNT) heterodimer pathway activates a series of genes involved in xenobiotic metabolism (including cytochrome P450). Although this pathway is not well studied in EOC, in breast, prostate, and oral squamous carcinomas, AHR is implicated in cell migration, invasion and metastasis [25-27] and has been proposed as a novel therapeutic target. JUND/JUNB is part of the AP-1 TF complex, which has been implicated in a broad array of cancer-relevant phenotypes including proliferation and apoptosis. Interestingly JUNB can interact with *BRCA1* [28], which, when mutated, confers a high risk of developing HGSOC.

There are limitations of this study. Since there are no TAD maps for EOC we used TADs defined in H1, a human embryonic stem cell line, as TADs are thought to be highly conserved across cell types [18]. However EOCs (particularly HGSOC) are highly genomically unstable, and contain many chromosomal rearrangements. Such rearrangements could disrupt TAD boundaries, removing or creating enhancer-target gene interactions. TAD maps and targeted or genome-wide chromosome conformation capture data (such as 4C or Hi-C) performed in the HeyA8 and IGROV1 cell lines would be required to confirm the predicted physical interactions between enhancer-associated PAX8 binding sites and the putative target genes. Another caveat is that we performed these studies in HeyA8 and IGROV1 cell lines because they have high expression of PAX8, form tumors in mice, and are models of non-subtype specific EOC. The results we present are thus generally applicable to all EOC, but may not reflect histotype-specific differences in the PAX8 cistrome.

The marked difference in PAX8 binding sites and target genes between the two cell lines may be due to the fact that they are likely different histotypes and, coming from different patients, the cells have different genetic and epigenetic backgrounds. PAX8 is overexpressed in many reproductive tract cancers and it is currently unclear if it has the same or different functions in these tumor types, and what the variation may be between patients. PAX8 activity is governed by the redox state of cells [29] and this may be contributing to the variation between HeyA8 and IGROV1 cell lines. However, the targets identified in the two different cell lines converged into common molecular pathways. As seen with many development-related oncogenes, several developmental pathways were enriched in the PAX8 cistrome: anatomical structural morphogenesis (which was common to both cell lines), tissue morphogenesis, positive regulation of programmed cell death, mesenchymal cell development, TAP63 signaling, Notch signaling and epithelial cell differentiation (which were cell line specific). In addition, supporting the *in vitro* and *in vivo* results, proliferation related pathways were identified: including DNA replication, G2M checkpoint, mitotic cell cycle process, DNA-directed DNA polymerase activity, regeneration, and KRAS signaling. Interestingly, 8 immune related pathways were found (cellular response to LPS, IKK/NFKB signaling, TNFA signaling via NFKB, complement system, TLR2 signaling, phagocytosis, IL1A production, NLR signaling), two of which were shared (cellular response to LPS and TNFA signaling via NFKB) between both cell lines. The immune system was also found to be associated with PAX8 in another study [PMID: 22531031]. Of the common DEGs between HeyA8 and IGROV1 knockdowns, *AJAP1, SIRPA, TGFBR2*, and *C20orf75* are cellular-adhesion related [30-33]. It is possible that PAX8 knockdown reduces the level of cellular adhesion, which may explain the reduced tumor burden in HeyA8 xenograft mice. This may also explain the decreased proliferation in anchorage dependent growth conditions.

To better characterize the PAX8 binding motif, we related the motif predicted through the ChIP-seq analyses to the predicted structure of the PAX8 protein in complex with DNA. PAX5 and PAX6 have been crystalized in complex with DNA [34, 35]. Due to its sequence similarity to PAX8, the PAX5/DNA complex was used as a template to generate a PAX8/DNA homology model. This revealed two subdomains, within the PAX8 DNA binding domain, that contact the major groove of DNA with greater specificity, joined by a linker domain with markedly lower DNA binding specificity. Importantly, the location of most critical bases in the motif could be predicted from the protein structure, which gives added weight to our predicted motif. Despite identifying the same motif in the two cell lines, there was a high degree of cell-type specificity in the genomic locations of the PAX8 binding sites and DEGs identified. The overlap was between 13%-22%, similar to the 26% of PAX8 binding sites in HGSOC cell lines that intersected the PAX8 binding sites in the FTSEC cell lines [17]. The small overlap in DEGs and PAX8 binding sites, but similar set of pathways altered, suggests that PAX8 has a highly cell-type specific regulatory network that focuses on a set of pathways involved in proliferation, migration/invasion and differentiation.

In conclusion, PAX8 is involved in transformed behavior of epithelial ovarian cancer cells *in vitro*, and suppresses tumor growth *in vivo*. We have established a global map of PAX8 direct and indirect regulatory targets. While it is clear that the PAX8 cistrome exhibits a high degree of cell-type specificity, analyses of deregulated pathways and co-factors converged on common molecular targets and partners that may represent important and much needed therapeutic targets for EOC.

## MATERIALS AND METHODS

### Analysis of TCGA data

TCGA microarray data were downloaded from The Cancer Genome Atlas portal and analyzed in ‘R’ using Bioconductor. Two-tailed paired T-Tests were performed to identify differences in PAX gene expression between HGSOCs and normal fallopian tube tissues.

### Immunohistochemistry

Immunohistochemistry (IHC) for PAX8 (antibody product number 10336, Proteintech) was performed on sections of normal ovaries and on a tissue microarray (TMA) containing around 160 ovarian carcinoma biopsies from patients with known outcome. Staining was confirmed on whole sections from another part of the tumour for ∼10% of the TMA biopsies. IHC staining intensity was used to assess PAX8 protein expression in each tissue (0=negative; 1=low; 2=medium; 3=high). Human tissues were used with the approval of the institutional review board at the University of Derby.

### Cell culture and PAX8 knockdown

IGROV1 (luciferase labelled) and HeyA8 ovarian cancer cells were cultured in Dulbecco’s Modified Eagle’s medium (Caisson) and RPMI base media (Lonza), respectively. Media were supplemented with 10% fetal bovine serum (FBS, Seradigm). Lentiviral supernatants containing individual short hairpin RNAs against *PAX8* or control shRNA was generated by co-transfection of HEK293T vectors with 3^rd^ generation packaging vectors and PAX8-shRNAs cloned into the pLKO.1 vector (Sigma Aldrich) (clone IDSs: NM_003466.3-772s21c1 (shPAX8_1), NM_03346.3-1070s21c1(shPAX8_2), NM_003466.2-784s1c1 (shPAX8_3), NM_003466.3-1917s21c1 (shPAX8_4)). HeyA8 and IGROV1 cells were infected by overnight lentiviral transduction and positive cells selected using 800 (HeyA8) or 200 (IGROV1) ng mL^-1^ puromycin (Sigma-Aldrich). Cell line authentication was performed on all cell lines using the Promega Powerplex 16HS Assay (performed at the University of Arizona Genetics Core facility). All cultures were confirmed to be free of *Mycoplasma* infections using a Mycoplasma specific PCR.

### RT-qPCR

RNA was harvested using the Quick-RNA MiniPrep kit (Zymo Research) and reverse transcribed to cDNA using the M-MLV reverse transcriptase (Promega) or the SuperScript III First-Strand Synthesis System (Invitrogen). Target gene expression was normalized to *ACTB* and *GAPDH* expression (Life Technologies), and relative expression calculated using the ∆∆Ct method. Probe information can be found in Supplementary Table 2.

### *In vitro* phenotypic assays

Growth curves were performed by plating 1-2 x10^5^ cells into triplicate P60 dishes and passaging and counting cells at regular intervals. Population doublings (PDs) were calculated using the following formula PD = log (final cell number/initial cell number)/log2, and cumulative population doublings plotted. For anchorage independent growth assays 1-3 x10^3^ cells were suspended in culture media containing 0.33% Noble Agar (Sigma Aldrich), plated on top of a base layer of culture media containing 0.6% Noble Agar. After 4 weeks colonies were stained by overnight incubation at 37°C with 1% p-iodonitrotetrazolium violet (Sigma) dissolved in 100% methanol (VWR). Colonies were visualised and counted by phase microscopy.

### *In vivo* tumorigenicity assays

Xenograft assays were performed under the approval and guidance of the University of Southern California Institutional Animal Care and Use Committee. 8 million cells were injected intra-peritoneally into 6-week old nu/nu mice (Simonsen Laboratories). Animals were sacrificed after 28 days and tumors were measured by digital caliper. After measurement tumors were fresh frozen for tissue sectioning.

### Microarray analyses

RNA was isolated in triplicate and gene expression microarray profiling performed using the Illumina HumanHT-12 v4 Expression BeadChips. Arrays were run at the University of Southern California Epigenome Core and University of California at Los Angeles Neuroscience Genomics Core, using standard protocols. Data analysis was performed using Partek Genomics Suite. For each cell line triplicate arrays for two independent knockdown clones were compared to control shRNA and parental samples.

### Chromatin immunoprecipitation sequencing

Chromatin immunoprecipitation sequencing (ChIP-seq) was performed based on the methods of Schmidt *et al.* [36]. Cells were fixed in 1% formaldehyde for 10 minutes, and quenched with glycine. Cells were harvested, lysed in a sarkosyl-containing buffer, and sonicated using the Covaris E220evolution Focused-Ultrasonicator. 10 μg of an antibody raised against PAX8 (NBP1-32440, Novus) or 5 μg of an antibody raised against lysine 27 acetylated histone 3 (H3K27ac) (C15410196, Diagenode) was incubated with 100 μg and 4ug, respectively, of chromatin at 4°C overnight. Blocked magnetic Dynabeads (Life Technologies) were then added to the antibody-lysate conjugates and incubated at 4°C for 4 hours with rotation. Beads were then washed with RIPA buffer and treated with RNase and proteinase K (both Qiagen). DNA was eluted from the beads in Tris-EDTA buffer and cleaned up using the QIAquick PCR Purification kit (QIAgen). For each cell line two independent immunoprecipitations and one input sample were submitted for next-generation sequencing at the USC Epigenome Core Facility.

### ChIP-seq data analysis

ChIP-seq data were processed using MACS2 [37] with p-value cutoff of 0.001. The smaller of input or signal was linearly scaled to the same depth as the larger dataset. In order to control the irreproducible discovery rate in ChIP-seq analysis, we used IDR [38] version 2.0 pipeline with a threshold of *p* < 0.05. After mapping to hg19 with BWA [39] and removing duplicates, there were >15 million high-quality reads for all ChIP-seq replicates. We used SPP [40] to calculate the cross-correlation QC metrics (Supplementary Tables 3 & 4) and MACS2 + IDR to obtain the rescue ratio and self-consistency ratio for each cell line. In addition, we used SPP+IDR for peak calling and compared with the MACS2+IDR peaks. We found that ∼94% of the peaks called by MACS2+IDR pipeline where included in the SPP+IDR pipeline, which gives us confidence to use the MACS2+IDR peaks for discovery of the PAX8 cistrome.

HeyA8 and IGROV1 have 21,563 (average length = ∼2.4 Kbp) and 15,030 (average length = ∼2 Kbp) H3K27ac enriched regions, respectively. We used HOMER [41] to broadly identify super enhancer regions using the H3K27ac ChIP-seq peaks for individual replicates. Then, to avoid inclusion of non-reproducible peaks in this analysis, we merged the MACS2+IDR H3K27ac peaks that overlap the super enhancer regions identified by HOMER, and used the MACS2+IDR/HOMER regions as the superenhancer regions for each cell line.

For motif discovery, we used the web application MEME-ChIP [42] using all PAX8 peaks for each cell line, individually. To associate the differentially expressed genes and the ChIP-seq PAX8 peaks, we used a set of topological association domains (TADs) for H1 embryonic stem cells [18].

Pathway enrichment analysis was performed with the web application Metascape [19], using a custom analysis including “Oncogenic signatures”, “GO Molecular Function”, “Canonical Pathways”, “GO Biological Processes”, Hallmark Gene Sets”, and “KEGG Pathway” with the default parameters.

## SUPPLEMENTARY MATERIALS TABLES








